# Effect of Mach number on droplet aerobreakup in shear stripping regime

**DOI:** 10.1007/s00348-020-03026-1

**Published:** 2020-08-09

**Authors:** Zhaoguang Wang, Thomas Hopfes, Marcus Giglmaier, Nikolaus A. Adams

**Affiliations:** Chair of Aerodynamics and Fluid Mechanics, Technical University of Munich, 85748 Garching, Germany

## Abstract

**Abstract:**

The present experimental study investigates the shear stripping breakup of single droplets in subsonic and supersonic gaseous flows. In contrast to most research that places emphasis on the Weber number (We), we focus on the individual effects exerted by flow Mach (*M*_∞_) and Reynolds numbers (Re). Millimeter-sized droplets made of either ethylene glycol or water are exposed to shock-induced flows. Shadowgraph and schlieren images of the breakup process are recorded by an ultra-high-speed camera. The experimental We is constrained at 1100, while *M*_∞_ is varied from 0.3 to 1.19 and Re from 2600 to 24,000. A systematic analysis of the experiment series reveals that the breakup pattern alters with *M*_∞_ although a constant We is maintained. The classical stripping behavior with fine mist shed from the peripheral sheet changes to rupture of multiple bags along the periphery at *M*_∞_ = 0.63, and further to stretching of ligament structures from the leeward surface at *M*_∞_ = 1.19. The corresponding breakup initiation is delayed and the resultant fragments are sized less uniformly and distributed over a narrower spread. In terms of the early-stage deformation, droplets experience less intense flattening and slower sheet growth at higher *M*_∞_. The change of Re introduces additional variations, but only to a minor extent.

**Graphical abstract:**

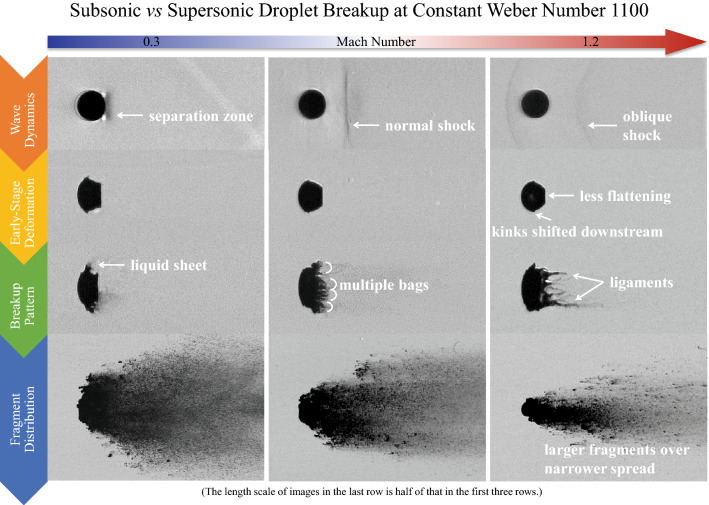

## Introduction

Droplet breakup, also termed secondary atomization, refers to the fragmentation of a droplet subjected to aerodynamic forces. This phenomenon is relevant in diverse applications, such as fuel injection (Reitz and Diwakar 1986), spray coatings (Mostaghimi et al. 2002) and metal powder production (Lagutkin et al. 2004). It has been widely recognized that the breakup morphology is primarily determined by the Weber number (We) and the Ohnesorge number (Oh) (Lane 1951; Hinze 1955):

1 $${\text{We}} = \rho_{g} u_{g}^{2} d_{0} / \sigma$$

2 $${\text{Oh}} = \mu_{d} / \sqrt {\rho_{d} d_{0} \sigma } ,$$

where *ρ*_g_ and *u*_g_ are the density and the velocity of the gas flow, and *d*_0_, *σ*, *μ*_d_ and *ρ*_d_ are the initial diameter, the surface tension, the dynamic viscosity and the density of the liquid droplet, respectively. The Weber number represents the ratio between the disruptive aerodynamic force and the restorative surface tension, and the Ohnesorge number compares the viscous force to the surface tension. According to Guildenbecher et al. (2009), the influence of liquid viscosity on the breakup regime diminishes when Oh drops below 0.1, leaving We as the dominant factor.

Several breakup mechanisms have been identified in the literature and are classified as bag breakup at 11 < We < 35 and stripping breakup at We > 80, with so-called multimode breakup in the intermediate range (Hsiang and Faeth 1995; Schmehl 2003). The bag breakup is conventionally understood as a result of the Rayleigh–Taylor instability developed at the droplet front (Joseph et al. 1999). However, some studies suggest different physical mechanisms, including the pressure imbalance between the front and rear side (Opfer et al. 2014), the stress repartition around the surface (Villermaux and Bossa 2009) and the structure of flow vortices in the wake (Inamura et al. 2009). The cause of the stripping breakup is also under debate. No agreement has been achieved on whether the viscous shear or the aerodynamic drag is the driving force. Correspondingly, the name of this regime varies among shear stripping (Ranger and Nicholls 1969), sheet thinning (Liu and Reitz 1997) and shear-induced entrainment (Theofanous and Li 2008). For the current work, we focus on the stripping breakup and adopt the concept proposed by Theofanous and Li (2008).

Although the breakup mechanism is mainly governed by the Weber number, other non-dimensional parameters influence the breakup behavior as well. Chou et al*.* (1997) conduct experiments with Ohnesorge numbers below 0.1, and observe larger micro-drops generated at higher Oh. Pilch and Erdman (1987) analyze the effect of Oh on the breakup time and conclude a consistent postponement of the breakup initiation as Oh increases. Lee and Reitz (2000) experimentally show that the liquid–gas density ratio (*ε* =  *ρ*_d_/*ρ*_g_) exerts negligible effects on the breakup process at values higher than 100. In numerical simulations by Kékesi et al*.* (2014); however, new breakup patterns appear for density ratios below 100. In terms of the flow Reynolds number (Re =  *ρ*_g_*u*_g_*d*_0_/*µ*_g_), the work of Liu and Reitz (1997) indicates that the breakup behavior is independent of Re when Re > 500. The dependence becomes important only in the Stokes flow (Aalburg et al. 2003) and in liquid–liquid breakup systems (Hsiang and Faeth 1995).

Another non-dimensional parameter, which is of significance but not fully explored, is the flow Mach number *M*_∞_. Most of preceding experiments are conducted at subsonic conditions, where the effect of the flow compressibility is marginal. However, with the recent development of supersonic combustion systems including pulse detonation engines (Kailasanath 2003), scramjet engines (Curran 2001) and supersonic gas atomizers (Anderson et al. 1991), droplet breakup in high-speed flows becomes of increasing importance. Dinh et al. (2003) and Theofanous et al. (2004) investigate various breakup regimes in a highly rarified flow at *M*_∞_ =  3. They find that the morphologies differ significantly from those categorized in subsonic flows and attribute the differences to changes in pressure fields. Ortiz et al. (2004) measure the drag coefficients of droplets in different airstreams and observe a rapid increase as the flow Mach number approaches supersonic conditions. Xiao et al. (2017) numerically simulate the deformation of droplets exposed to supersonic flows and conclude that the onset of breakup is postponed compared to subsonic cases. Igra and Takayama (2003) and Meng and Colonius (2015) conduct experimental and numerical research on water column breakup in high-speed flows, respectively. Both works quantitatively show a slower temporal increase of the cross-stream diameter at higher *M*_∞_.

Although the abovementioned research reveals distinct breakup features in supersonic flows, the experimental database addressing the effect of *M*_∞_ is rather limited. Moreover, a change of *M*_∞_ in experiments is commonly accompanied with a change of Re, which renders the independent investigation of *M*_∞_ difficult. In the present work, we constrain the Weber number at 1100 and decouple the correlation between *M*_∞_ and Re by applying different liquid–gas combinations and carefully choosing operating conditions. This creates a test matrix which allows us to study the effects of *M*_∞_ and Re individually.

## Experimental setup

**Fig. 1:**
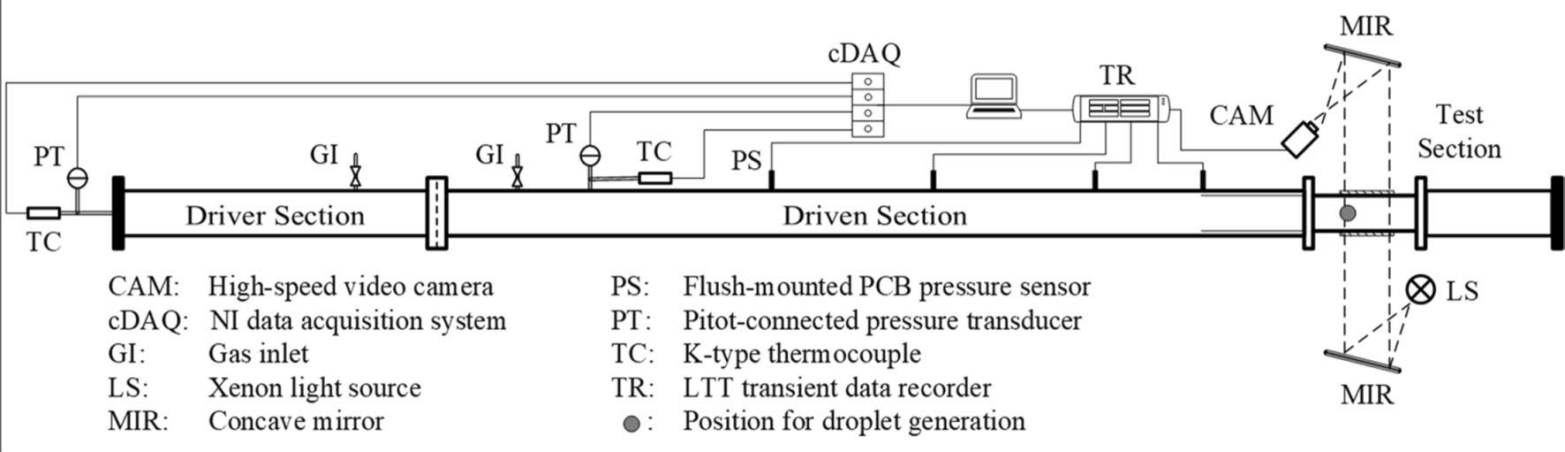
Layout of the shock tube and the measurement system

The layout of the shock tube used for the current experiments is depicted in Fig. 1. The tube, which has an overall length of 24 m and an inner diameter of 290 mm, consists of three segments: the driver, the driven and the test sections. A cookie cutter is installed in front of the test section to remove the boundary flows and to contract the cross section to a 190 × 190 mm^2^ square. In the experiments, a 0.15 mm-thick Mylar diaphragm is placed between the driver and driven sections and in contact with a pair of 0.1 mm-thick crossed NiCr heating wires. Each section of the shock tube is first filled to pressure levels corresponding to desired flow conditions. Then, a single droplet is produced in the test section by expelling liquids through a hypodermic needle with an outer diameter of 0.9 mm. The droplet falls through a pair of aligned laser emitter and receiver. This triggers the rupture of the Mylar diaphragm by supplying an electric current of 3 A to the heating wires. Subsequently, a planar shock wave forms, propagates towards the downstream test section and induces a flow with uniform flow conditions.

**Fig. 2:**
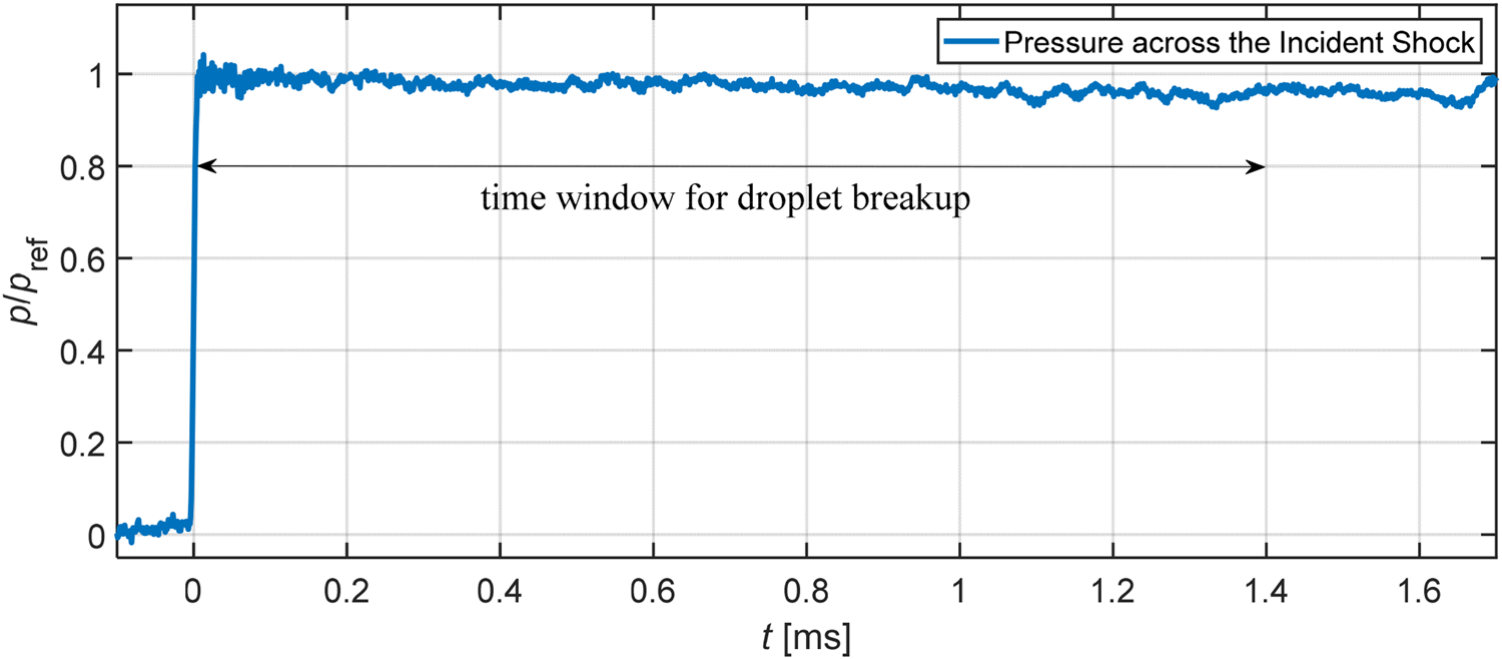
Step-wise pressure rise across the incident shock measured in the test section

The pressure variation along the shock tube is measured by flush-mounted PCB Piezotronics ICP^®^ fast-response pressure sensors. The measured signals are acquired by a LTT transient data recorder at a sampling frequency of 1 MHz. We calculate the shock velocity based on the time lag between moments when the incident shock passes two pressure sensors ahead of the test section. The combination of the shock velocity and initial pre-shock conditions yields post-shock flow properties based on moving shock relations. Another sensor in the test section measures the pressure rise across the incident shock. A representative pressure signal normalized against the theoretical post-shock pressure is provided in Fig. 2. The shock-induced freestream remains steady over 1.6 ms which well covers the investigated period of droplet breakup (maximally 1.4 ms). The slight decline in the pressure signal is attributed to the growth of the boundary layer as well as the nature of the piezoelectric sensors. This pressure signal also serves as a trigger for the image recording.

As for the flow visualization, a Shimadzu HyperVision HPV-X ultra-high-speed camera is integrated in a Z-type shadowgraph/schlieren photography system. The camera records 128 consecutive images with a resolution of 0.087 mm/pixel at a framing rate of 100 kfps.

**Fig. 3:**
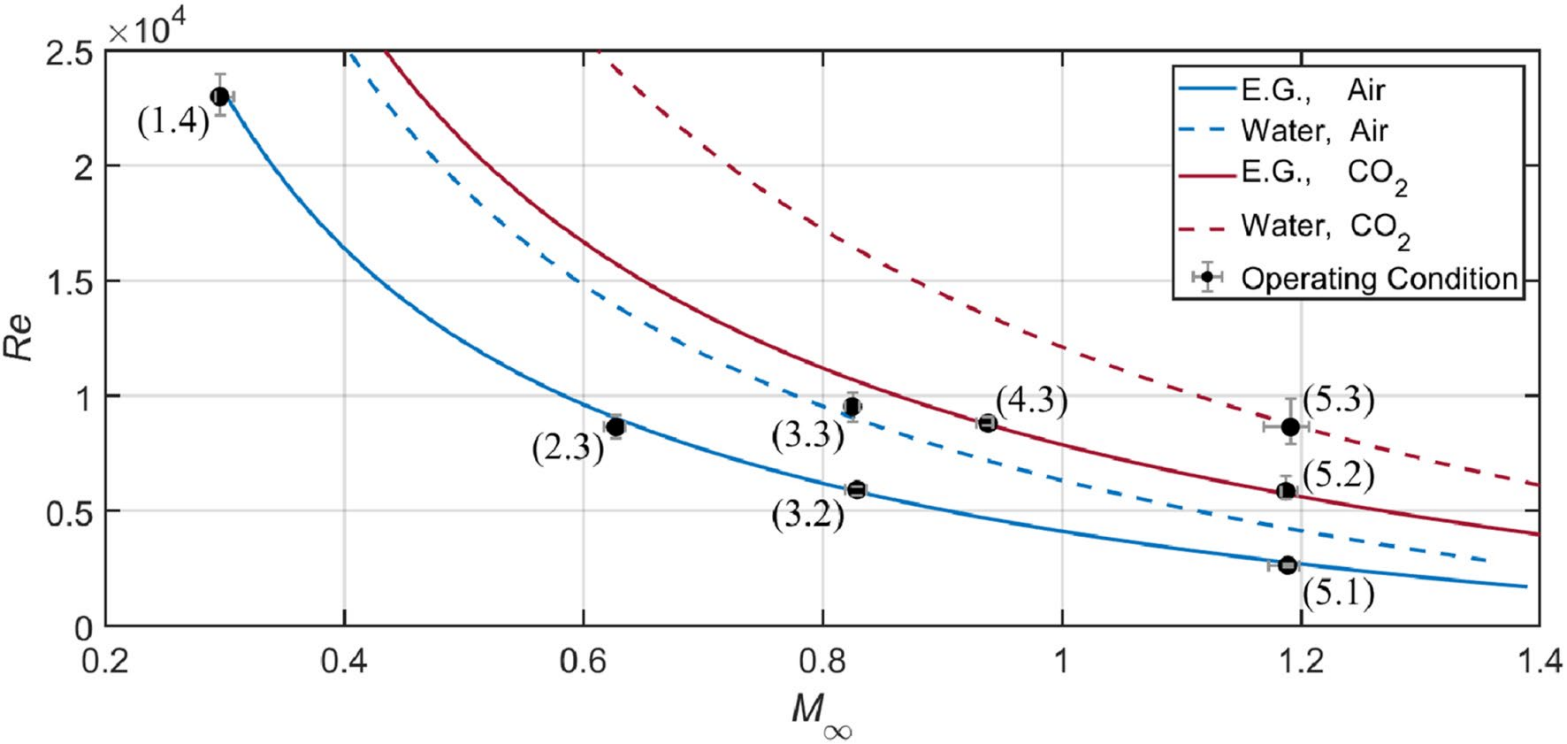
Inversely proportional correlation between *M*_*α*_ and Re at We = 1100. Each line corresponds to a certain liquid–gas combination. The eight operating conditions are labeled as *i*·*j*, with *i* and *j* representing the relative magnitudes of *M*_*α*_ and Re respectively. The associated error bars stand for the ranges of conditions from repeated experiments and are magnified twice in the plot for a clearer display

For the present experiments, the combinations of two liquids (ethylene glycol and water) and two gases (air and CO_2_) are exploited to analyze the effects of *M*_∞_ and Re independently at a constant We. The Weber number, which is constrained at 1100 with a standard deviation of 50, lies within the stripping breakup regime and allows a wide variation of *M*_∞_ and Re. Figure 3 shows the inversely proportional correlation between *M*_∞_ and Re for different liquid–gas combinations. The eight labelled points represent the operating conditions selected for current experiments, and the associated error bars (magnified twice in the plot) stand for the ranges of *M*_∞_ and Re from repeated experiments. The eight operating conditions are numbered as *i*·*j*, where the values of *i* and *j* correspond to the relative magnitudes of *M*_∞_ and Re*,* respectively. Detailed parameters averaged from repeated experiments at each operating condition are summarized in Table 1.

**Table 1:** Operating conditions averaged from repeated experiments

	Liquid	Gas	We	Oh	Re	*M*_*∞*_	*ρd/ρg*	*μd/μg*
Case 1.4	E.G	Air	1060	0.042	2.4e4	0.3	7.1e2	796
Case 2.3	E.G	Air	1056	0.044	8.6e3	0.63	3.6e3	697
Case 3.2	E.G	Air	1120	0.043	5.9e3	0.83	7.1e3	635
Case 5.1	E.G	Air	1050	0.044	2.6e3	1.19	2.1e4	508
Case 3.3	Water	Air	1160	0.002	9.5e3	0.83	4.5e3	35
Case 4.3	E.G	CO_2_	1100	0.044	8.8e3	0.94	4.7e3	802
Case 5.2	E.G	CO2	1120	0.043	5.9e3	1.19	9.0e3	713
Case 5.3	Water	CO_2_	1080	0.002	8.6e3	1.19	6.1e3	39

For water (*ρ*_*d*_  = 998 kg/m^3^, *σ*  = 7.28e-2 N/m, *μ*_*d*_ = 8.9e-4 kg/m s), the average droplet diameter is 3.1 mm, resulting in an Oh of 0.002. For ethylene glycol (*ρ*_*d*_  = 1113 kg/m^3^, *σ* = 4.73e-2 N/m, *μ*_*d*_ = 1.61e-2 kg/m s), the lower surface tension leads to smaller droplets with an average diameter of 2.6 mm and the considerably higher viscosity yields an Oh of 0.043. Concerning all the experiments, the freestream Mach number *M*_∞_ varies from subsonic (0.3) to supersonic (1.19) levels and the droplet diameter-based flow Reynolds number Re ranges over an order of magnitude (from 2.6e3 to 2.4e4). The liquid–gas density and viscosity ratios are also provided in Table 1 for completeness.

Case 1.4, 2.3, 3.2 and 5.1 are conducted with the same liquid and gas. Comparisons between them highlight the overall influences of parameters other than We and Oh. Moreover, Case 2.3, 4.3 and 5.3 have comparable Re but different *M*_∞_, while Case 5.1, 5.2, and 5.3 share the same *M*_∞_ but changing Re. Comparisons of the former and of the latter group shed light on the individual role played by *M*_∞_ and Re, respectively.

## Results and discussion

**Fig. 4:**
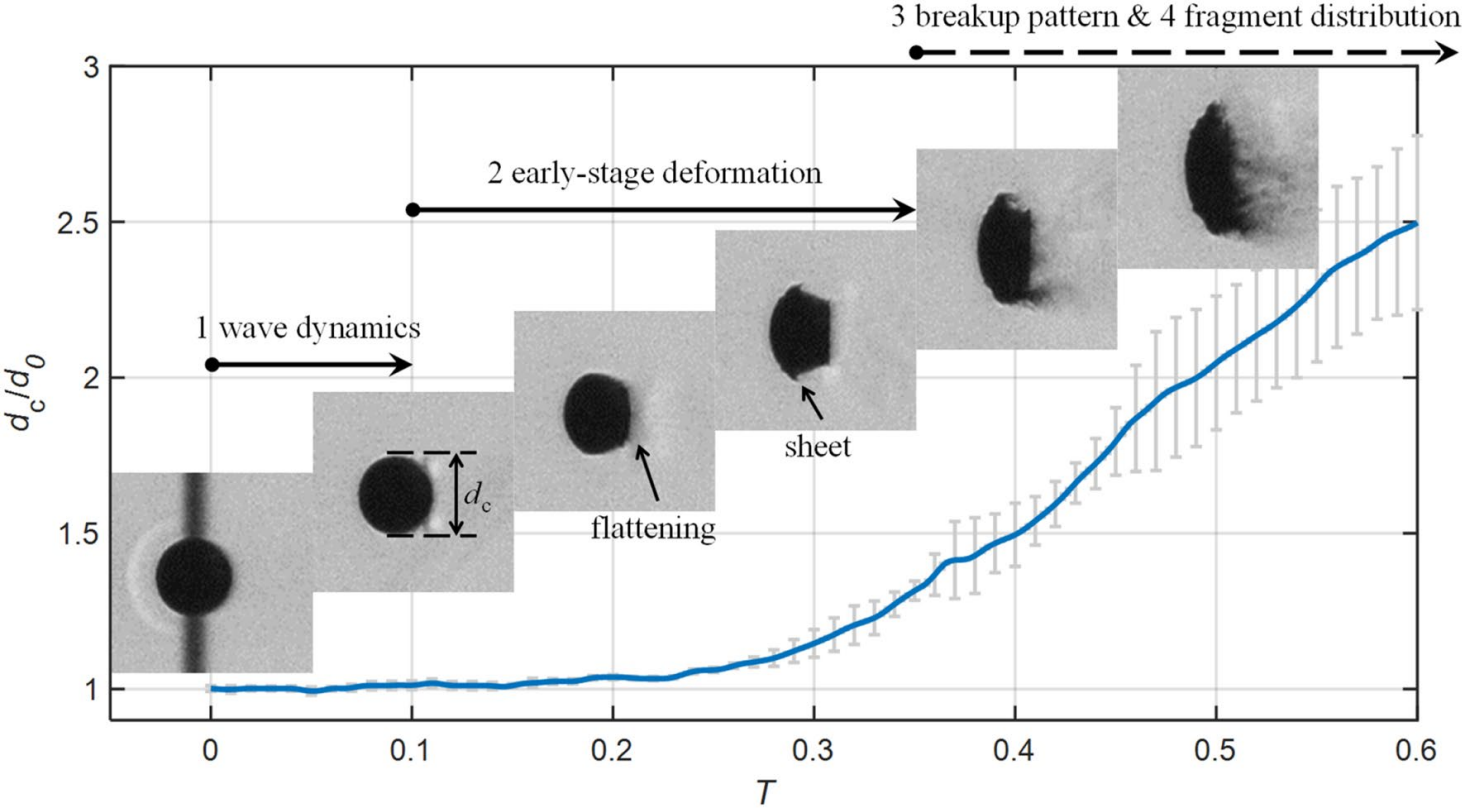
Breakup patterns and cross-stream diameter variation of an ethylene glycol droplet at *M*_*α*_ = 0.3 (Case 1.4). The error bars represent uncertainties of *d*c calculated from four repeated experiments

A brief overview of the typical stripping breakup process in subsonic flows (Case 1.4, *M*_∞_ = 0.3, liquid: ethylene glycol, gas: air) is provided in Fig. 4. Here, the experimental time *t* is regarded as zero at the moment when the incident shock impacts on the droplet. Furthermore, *t* is normalized against the characteristic transport time derived by Ranger and Nicholls (1969) based on droplet deformation in incompressible flows, yielding the dimensionless time *T* as

3 $$T = t \cdot u_{g} / \left( {d_{0} \sqrt {\rho_{d} /\rho_{g} } } \right),$$

The time scaling in Eq. (3) does not account for the compressibility effects, which govern the droplet breakup in the current study. Nevertheless, this scaling is used to present the results in a consistent and comparable way with previous literature. Presented images are processed with subtraction of the background noise, contrast stretching and super resolution using MATLAB’s Very Deep Super-Resolution convolutional neural network (Kim et al. 2016).

The entire breakup process is divided into four stages. It starts with the shock-droplet interaction which establishes a flow field resembling that around a solid sphere (*T* < 0.1). Then, the droplet is flattened along the streamwise direction (*T* = 0.2) and a liquid sheet emerges at the equator (*T* = 0.3). The rupture of the sheet indicates the breakup initiation (*T* = 0.4) and the coherent body is continuously eroded at the periphery afterwards. The last stage is achieved when the whole droplet disintegrates into fragments distributed widely in the flow field (out of the time window shown in Fig. 4).

Figure 4 also presents the quantitative change of the droplet cross-stream diameter *d*_*c*_. The error bar represents the uncertainty (90% confidence level based on the Student’s *t*-distribution) calculated from four repeated experiments. Before the onset of breakup (*T* < 0.37), the increase of *d*_*c*_ indicates the flattening of the intact body and the associated uncertainty is low. Once the breakup starts, the intact body is shadowed by the fine mist. The interpretation of *d*_*c*_ changes to a description of the spatial distribution of liquid fragments. The corresponding uncertainty significantly increases as micro-drops are more sensitive to local flow disturbance than the coherent body. Considering the uncertainty levels are similar for all cases, the error bars are omitted in the following plots for the sake of brevity.

In following sections, we compare cases with variations in flow Mach and Reynolds numbers with respect to each stage of the breakup process, namely wave dynamics, early-stage deformation, breakup patterns, and fragment sizes and spatial distributions. The main comparison is made between Case 1.4, 2.3, 3.2 and 5.1 all of which employ the same liquid and gas, to illustrate the overall tendency. This is further complemented by comparisons between constant-Re and constant-*M*_∞_ cases to highlight the individual effects of *M*_∞_ and Re, respectively. The discussion is concluded with a brief analysis of the influence of Oh.

### Wave dynamics

**Fig. 5:**
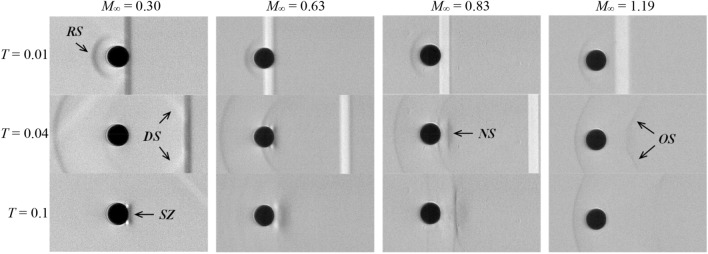
Wave dynamics around ethylene glycol droplets in air. The operating conditions from left to right are Case 1.4, 2.3, 3.2 and 5.1. *RS* reflected shock, *DS* diffracted shock, *SZ* separation zone, *NS* normal shock, *OS* oblique shock)

Figure 5 presents a visualization of wave dynamics surrounding ethylene glycol droplets in airstreams after the impact of the incident shock for Case 1.4, 2.3, 3.2 and 5.1. At *M*_∞_ = 0.3, typical features including the reflected shock (RS) at the windward surface, the diffracted shock (DS) enclosing the droplet and separation zones (SZ) attached at the rear are clearly identified. These characteristics are in good agreement with the experimental and numerical results of water column breakup from Igra and Takayama (2001) and Meng and Colonius (2015).

The main change at *M*_∞_ = 0.63 is that the separation zone behind the droplet extends over a wider region. As the freestream speed enters the transonic range (*M*_∞_ = 0.83), a normal shock (NS) appears behind the droplet and fluctuates slightly around the displayed location. This suggests that the surrounding flow accelerates to supersonic conditions as it bypasses the droplet. For the case at *M*_∞_ = 1.19, an oblique shock cone (OS) appears, stretching downstream over a broad region. Meanwhile, the reflected shock ahead of the droplet settles as a detached bow shock.

Considering the droplet does not undergo noticeable deformation during the short period of shock-droplet interaction, the surrounding pressure field is expected to resemble that around a solid sphere, which is also implied by the wave patterns presented in Fig. 5. As *M*_∞_ changes, the pressure imposed on the spherical surface differs significantly (Charters and Thomas 1945; Bailey and Haitt 1972). These differences are held accountable for the more pronounced distinctions in succeeding deformation and breakup processes (Hanson et al. 1963).

**Fig. 6:**
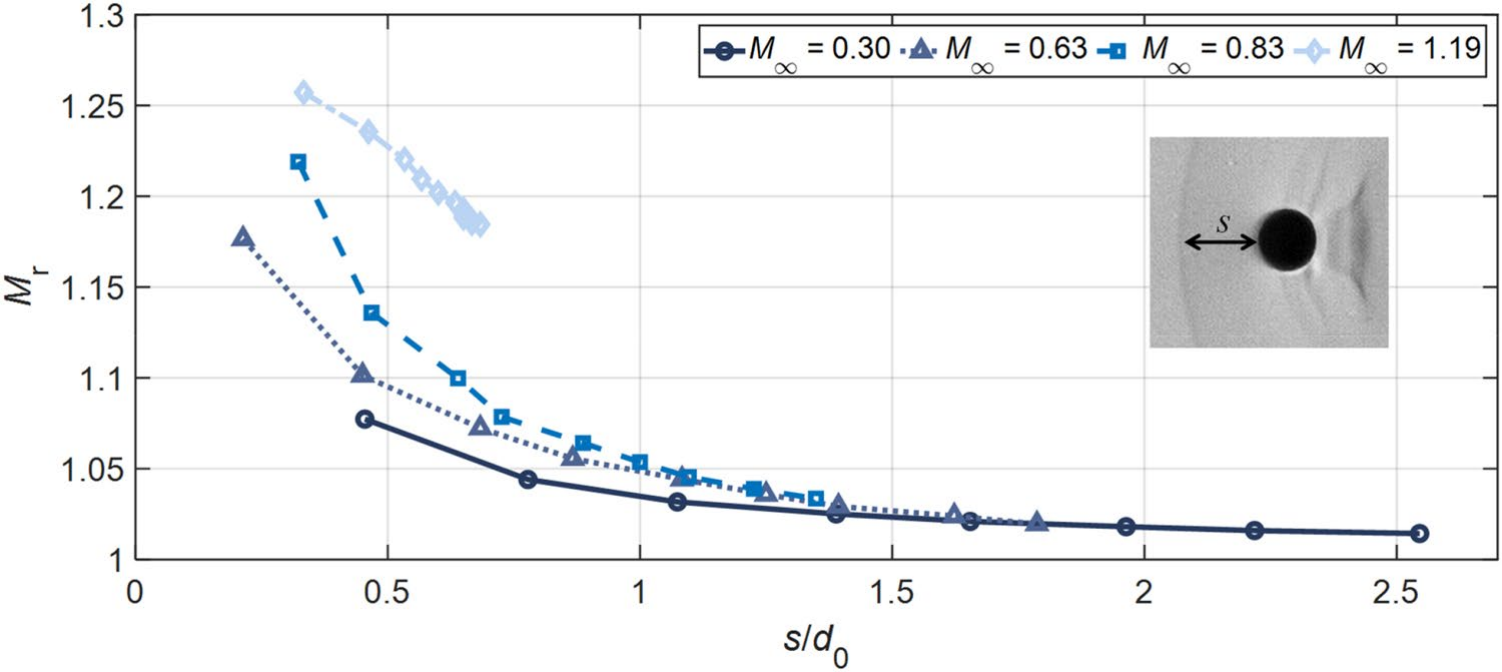
Mach number of the reflected shock relative to the freestream flow (*M*_*r*_) with respect to the normalized distance from the reflected shock to the droplet front (*s*/*d*_0_) for Case 1.4, 2.3, 3.2, 4.3 and 5.1

The travelling velocity of the reflected shock is plotted in Fig. 6 with respect to the position relative to the droplet front. The distance *s* between the reflected shock and the droplet leading edge is normalized by the droplet diameter *d*_0_. The Mach number of the reflected shock relative to the freestream flow is calculated as *M*_r_ = (d*s*/d*t* + *u*_g_)/*a*, where *a* is the speed of sound in the freestream. For cases with *M*_∞_ < 1, the reflected shock decays to sonic waves, and the corresponding *M*_*r*_ falls towards 1. For the supersonic case with *M*_∞_ = 1.19, however, the decreasing *M*_*r*_ settles at *M*_∞_ and the normalized distance *s*/*d*_0_ is stabilized around 0.65 which matches the value measured for a solid sphere at similar conditions (Liepmann and Roshko 2001).

### Early-stage deformation

**Fig. 7:**
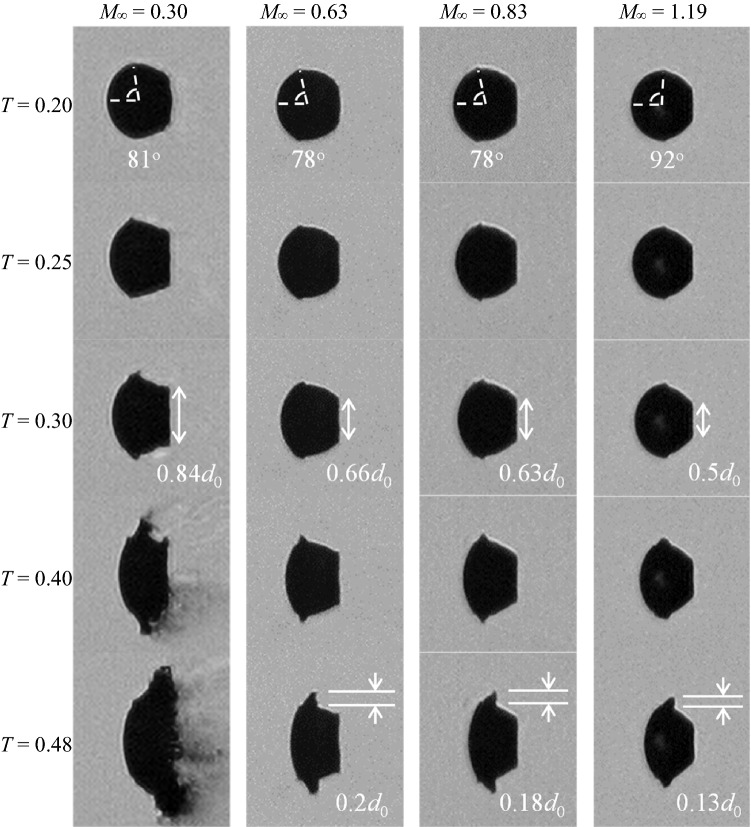
Droplet flattening and development of liquid sheets (left to right: Case 1.4, 2.3, 3.2 and 5.1)

The early-stage droplet deformation for subsonic and supersonic cases is compared in Fig. 7. As described in Fig. 4, the droplet at *M*_∞_ = 0.3 exhibits typical deformation features such as flattening at frontal and rear surfaces and stretching of the liquid sheet around the equator. Generally, droplets at higher-*M*_∞_ follow similar deformation patterns, but a thorough examination reveals noticeable distinctions that are summarized as follows:

- the liquid sheet emerges further downstream in the supersonic flow (*T* = 0.20);
- the flattening of the leeward surface becomes weaker as *M*_∞_ increases (*T* = 0.30);
- the liquid sheet grows more rapidly at lower *M*_∞_ (*T* = 0.48).

As the windward surface of the droplets gets flattened, kinks form around the droplet equator at *T* = 0.2. These kinks are the origins of the subsequent development of liquid sheets. In subsonic flows, the kink is located ahead of the equatorial plane and the angle of inclination of the line connecting the kink to the droplet center is approximately 80° with respect to the flow direction. However, the position of the kink is shifted considerably downstream in the supersonic flow and the corresponding angle of inclination is 92°. The kink locations are in good accordance with the trajectory of the separation point at the surface of a solid sphere measured by Charters and Thomas (1945). In their work, the separation point stays with an angle of inclination between 70° and 80° at subsonic conditions and drifts downstream beyond 90° at *M*_*∞*_ = 1.2.

During the period shown in Fig. 7, the leeward surface of the droplet is continuously flattened. The extent of the flattened area at *T* = 0.3 is estimated relative to the initial droplet diameter. As *M*_*∞*_ increases from 0.3 to 1.19, the corresponding flattened area shrinks from 0.84*d*_0_ to 0.5*d*_0_. This could be associated to the change of the pressure imposed on the droplet rear at different flow conditions. Karyagin et al. (1991) experimentally measure the pressure distribution over the surface of a sphere, and observe that the pressure at the rear surface drops consistently as *M*_*∞*_ increases. The same trend is also reported in the numerical work by Nagata et al. (2016).

**Fig. 8:**
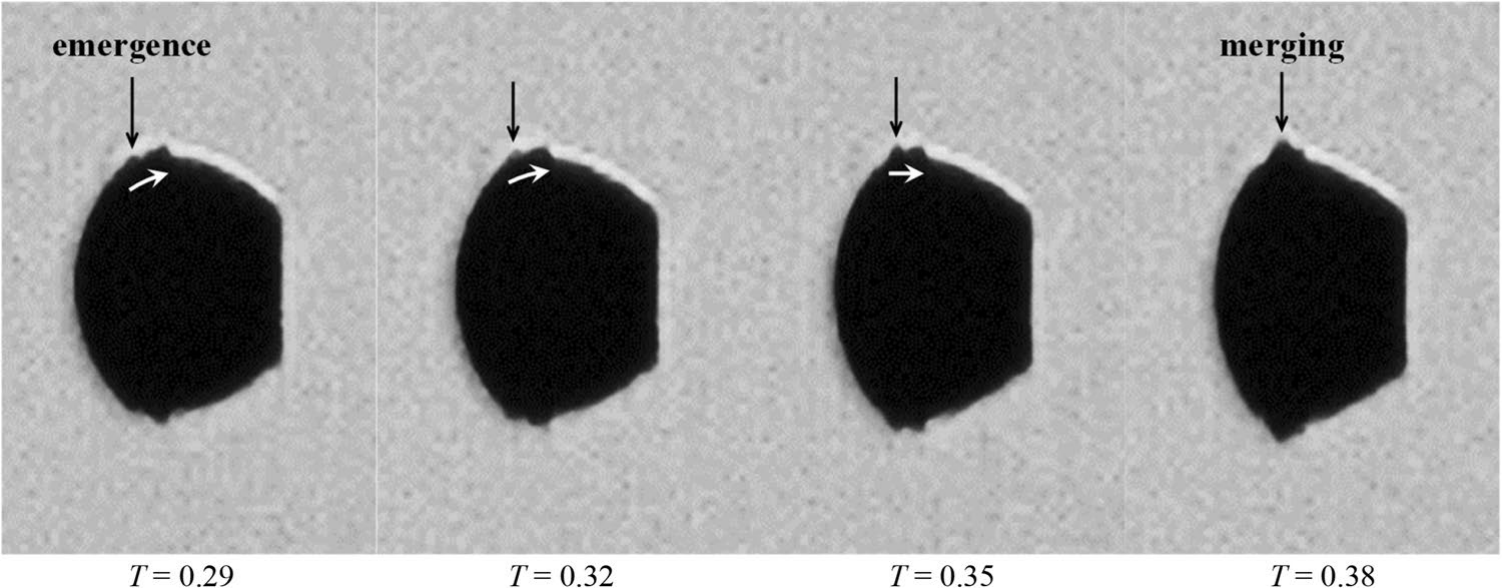
Emergence, propagation and merging of surface waves at the droplet periphery (Case 3.2, *M*_*∞*_ = 0.83). Dark and white arrows indicate locations and movement directions of the surface waves, respectively

By *T* = 0.48, the liquid sheets for all cases have grown to considerable sizes and stretched out radially from the main body. Similarly to the experimental observation by Theofanous et al. (2012) and the numerical analyses by Jalaal and Mehravaran (2014), the growth of the liquid sheets is enhanced by the emergence of propagative waves at the droplet surface, as shown in Fig. 8. These surface waves are induced by Kelvin–Helmholtz instabilities at the windward surface, where the liquid–gas interface suffers strong shear. The waves are transported towards the equator under drag forces and then merge with the preceding waves.

Papamoschou and Roshko (1988) point out that increasing the flow Mach number reduces the growth rate of the shear layer between two streams rapidly, because the associated compressibility effect tends to stabilize the flow disturbance. Moreover, according to Liepmann and Roshko (2001) and Nayfeh and Saric (1971), the pressure distribution around small-scale waves is out of phase with the wave profile in supersonic flows and thus suppresses the development of instabilities. Consequently, the growth rate of the liquid sheet is lower at higher *M*_*∞*_. This explains the observation at *T* = 0.48 in Fig. 7 that the liquid sheet becomes smaller as *M*_*∞*_ increases.

**Fig. 9:**
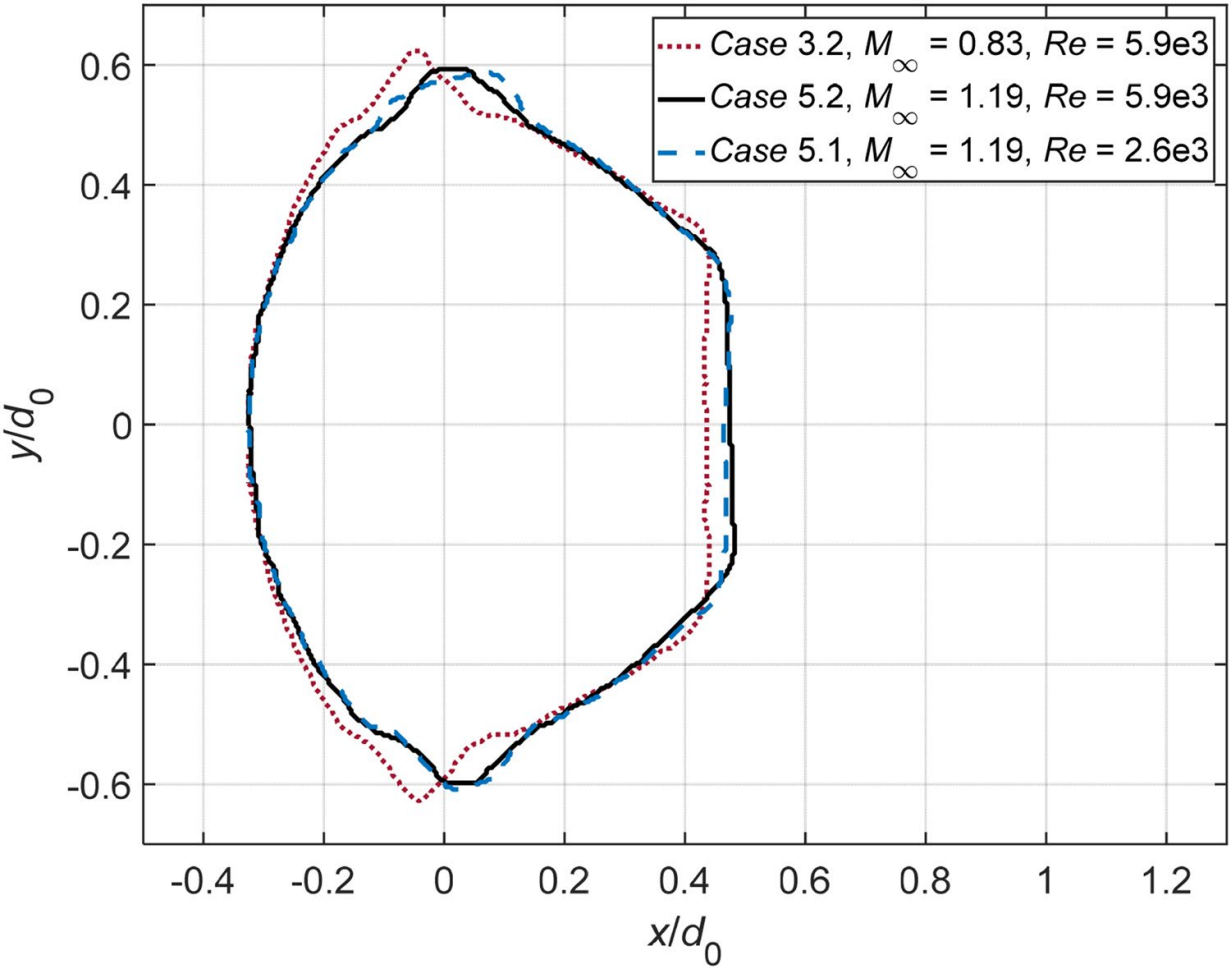
Droplet outlines of Case 3.2, 5.2 and 5.1 at *T* = 0.4. The mass center of the initial droplet lies at (0, 0)

Figure 9 compares the droplet contours at *T* = 0.4 between Case 3.2, 5.2 and 5.1. The dimensions are normalized against the initial droplet diameter, and the origin represents the position of the initial droplet center. Case 5.2 has the same *M*_*∞*_ as Case 5.1, and shares a comparable Re with Case 3.2. The resemblance of the droplet contours between Case 5.2 and Case 5.1 indicates that Re exerts negligible effects on the early-stage deformation, while *M*_*∞*_ plays a critical role in determining the flattening intensity and the sheet development.

To further quantify the droplet deformation, streamwise displacements of the leading edge, the mass center and the trailing edge are measured. These parameters are of particular interest for numerical validations.

**Fig. 10:**
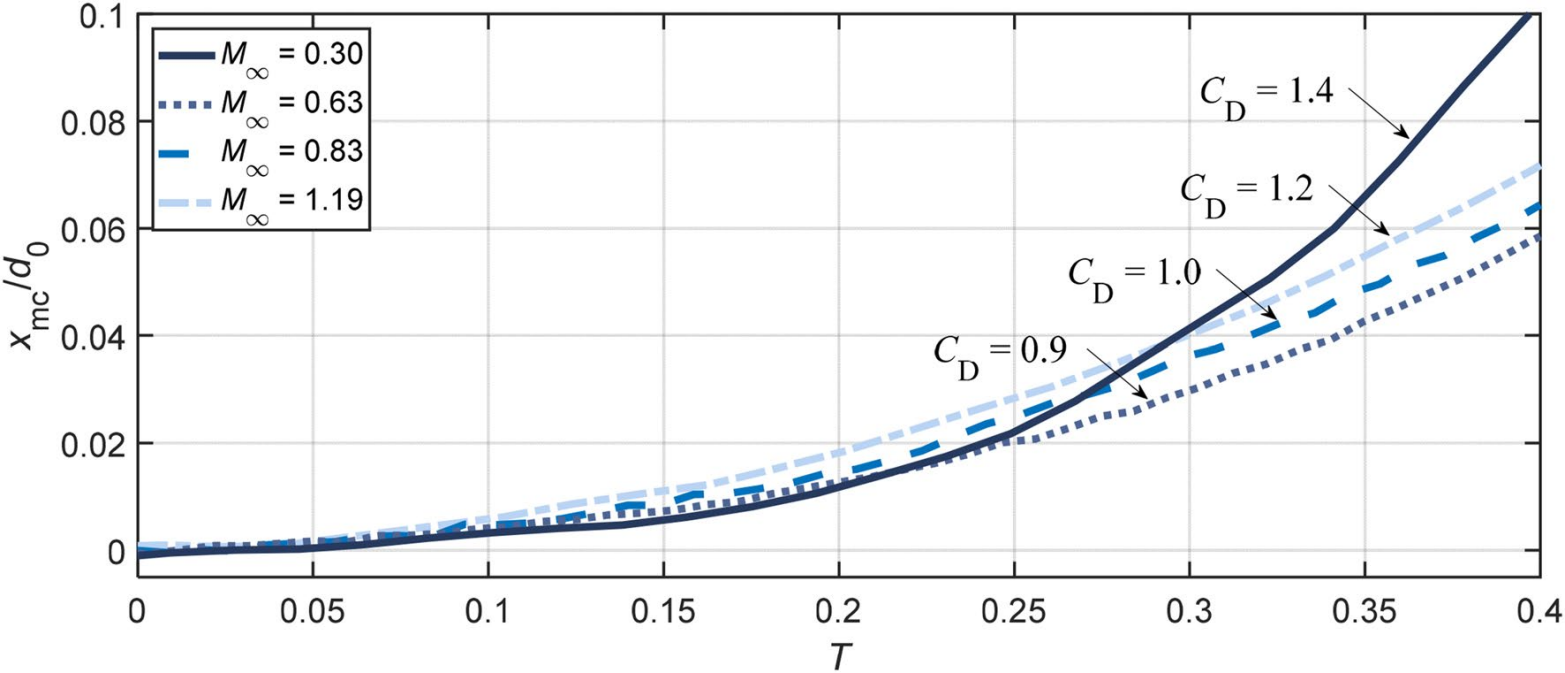
Streamwise displacement of the mass center for Case 1.4, 2.3, 3.2 and 5.1. Initial droplet center lies at *x* = 0. The plotted data are averaged values from four repeated experiments

The position of the mass center, which is calculated with the assumption that the droplet cross section normal to the flow direction is axisymmetric, is plotted in Fig. 10. For all cases, the trajectory of the droplet mass center approximates to be parabolic over the shown period. The drag coefficient CD is estimated by fitting the data into the relation xmc/*d*_0_ = 3/8CDT2 derived by Ranger and Nicholls (1969). The subsonic case at *M*_*∞*_ = 0.3 experiences a drastic acceleration around *T* = 0.25. This results from the rapid growth of the cross-stream diameter, as shown in Fig. 7, and yields a relatively high CD of 1.4. For the other three cases that share comparable cross-stream diameters before *T* = 0.4, the streamwise drift of the droplet mass center is faster at higher *M*_*∞*_. The corresponding drag coefficients are calculated to be 0.9 for *M*_*∞*_ = 0.63, 1.0 for *M*_*∞*_ = 0.83 and 1.2 for *M*_*∞*_ = 1.19. This trend agrees with the drag coefficients for a solid sphere measured by Bailey and Haitt (1972) and Charters and Thomas (1945), but the values are much higher due to the droplet flattening.

**Fig. 11:**
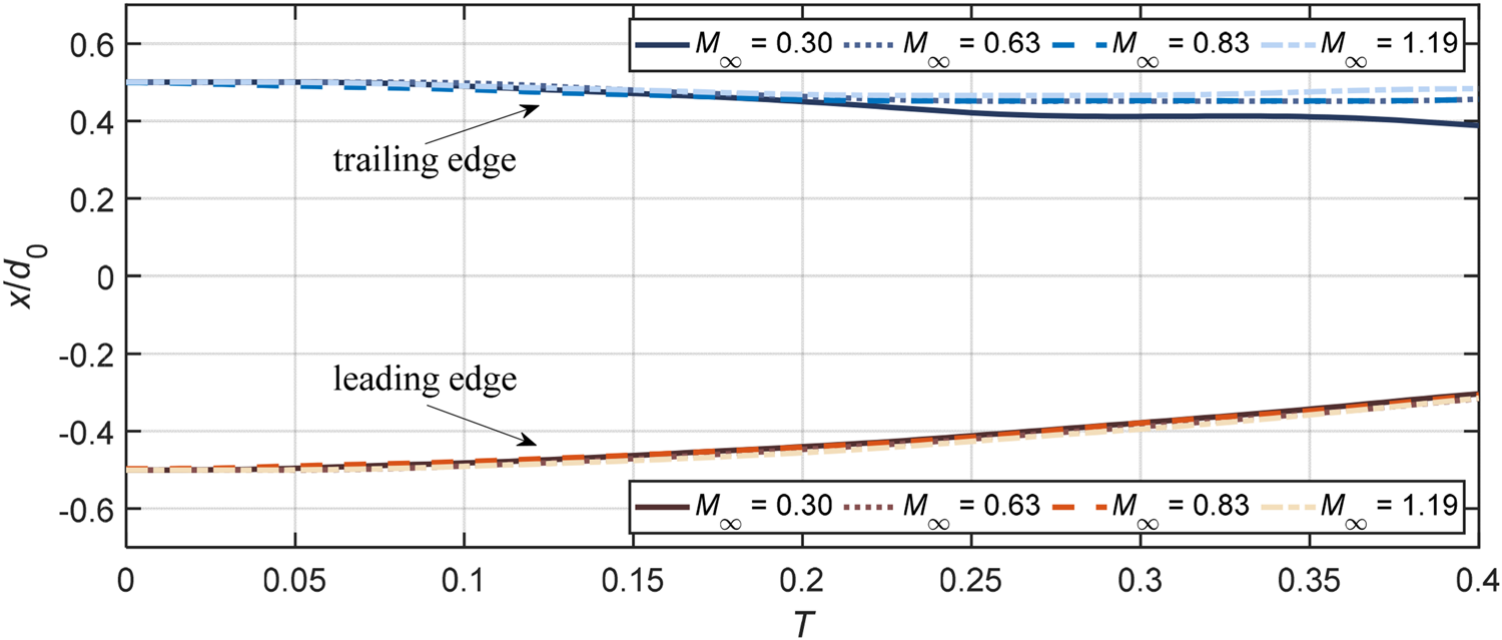
Streamwise displacement of the leading edge and the trailing edge for Case 1.4, 2.3, 3.2 and 5.1. Initial droplet center lies at *x* = 0. The plotted data are averaged values from four repeated experiments

Figure 11 compares the streamwise displacements of the leading edge and the trailing edge at different flow conditions. It is noteworthy that the difference of the leading edge shift is negligible among all present cases. Therefore, as also stated by Theofanous (2011), the displacement of the leading edge is not a proper parameter to represent the drag. In terms of the trailing edge, the displacement shows certain degrees of variations between cases and becomes smaller at higher *M*_*∞*_. This tendency is consistent with the observation in Fig. 7 that the flattening at the leeward surface is weakened as *M*_*∞*_ increases.

### Breakup patterns

**Fig. 12:**
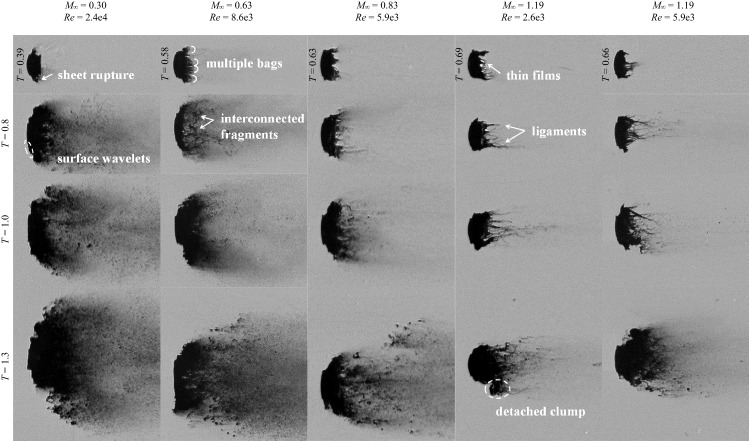
Droplet breakup patterns (left to right: Case 1.4, 2.3, 3.2, 5.1 and 5.2). The top row corresponds to the breakup initiation

Breakup patterns of droplets at different flow conditions are displayed in Fig. 12. The first row represents the individual breakup initiation which is defined as the onset of the formation of liquid fragments. Each of the remaining rows corresponds to a specific time moment. Although the breakup process shows certain degrees of chaotic behavior (Hardalupas and Whitelaw 1994; Engelbert et al. 1995), the features discussed in the following are consistently observed in repeated experiments. Generally speaking, three types of breakup patterns are categorized:

- fragmentation of the liquid sheet (*M*_∞_ = 0.3);
- development of multiple bags along the periphery (*M*_∞_ = 0.63);
- formation of streamwise ligaments in the wake (*M*_∞_ = 1.19).

The droplet at *M*_∞_ = 0.3 experiences a typical stripping breakup initiated with the fracture of the liquid sheet. Afterwards, the windward surface is continuously flattened and expands over an increasingly broad region. At regions encircling the smooth front, small-scale waves generated by Kelvin–Helmholtz instabilities appear. These waves propagate towards the periphery peeling micro-drops off from the droplet. The micro-drops are entrained in the flow and distributed widely in the cross-stream direction.

**Fig. 13:**
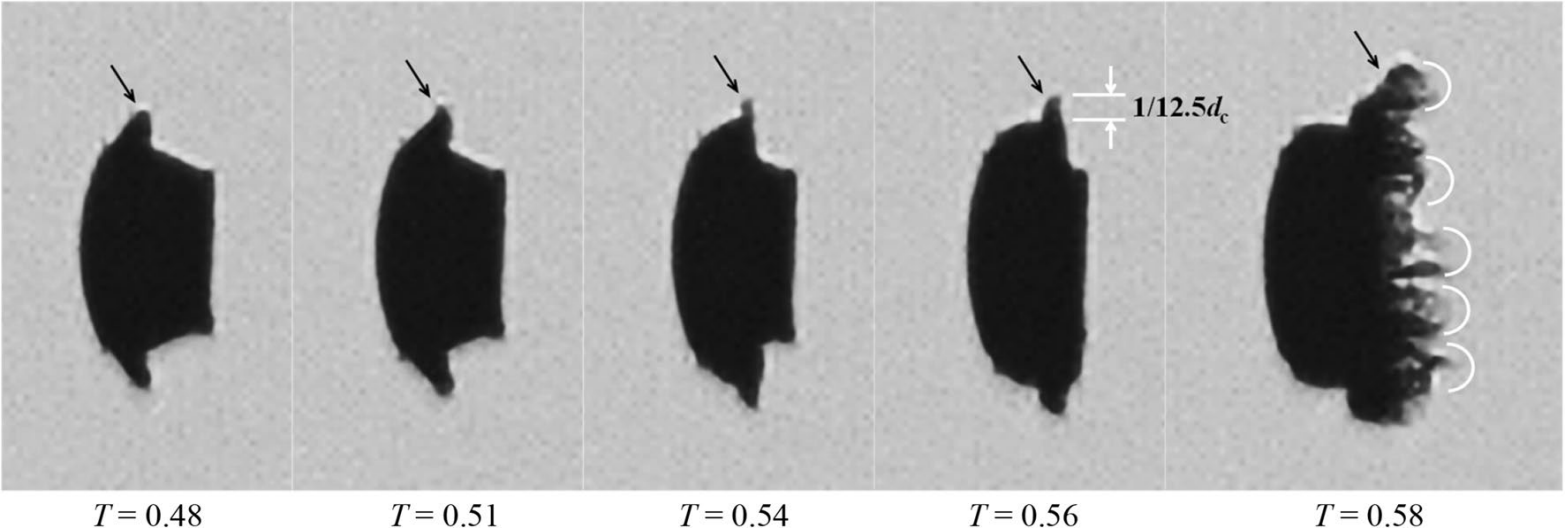
Straightening of the droplet rim (indicated by the arrows) and formation of multiple bags along the periphery for Case 2.3 at *M*_*∞*_ = 0.63

The breakup pattern is altered as the flow Mach number increases. At *M*_∞_ = 0.63, the breakup onset is not indicated by entrainment of micro-drops, but by the formation of multiple bags along the periphery. Figure 13 shows the evolution from the bending of the liquid sheet to the inflation of the multiple bags. As the sheet extends downstream, the peripheral region is straightened to directly face the freestream flow (*T* = 0.54). Then, multiple bags form along the rim and inflate rapidly (*T* = 0.59). These bags rupture into fine mist and the rings that the bags are attached to disintegrate into larger interconnected fragments as indicated at *T* = 0.8 in Fig. 12. The remaining coherent body of the droplet deforms into a crescent shape and the succeeding breakup process resembles the typical stripping pattern.

According to Theofanous et al*.* (2004) and Guildenbecher et al*.* (2009), the wave number *n* of the fastest-growing wave induced by Rayleigh–Taylor instabilities at the droplet front can be calculated with the following relation:

4 $$n = \frac{1}{2\pi }\left( {\frac{{d_{c} }}{{d_{0} }}} \right)^{2} \sqrt {C_{D} \cdot \text{We}}$$

For Case 2.3 at *T* = 0.56, *d*_*c*_ is 1.53 times of the initial diameter *d*_0_ (see Fig. 15) and the average value of *C*_D_ is 0.9 (see Fig. 10), which yields *n* = 11.7. The actual value of *n* should be higher considering the real-time *C*_*D*_ is growing over time. Nevertheless, the calculated wave number agrees well with the observation in Fig. 13 that the straightened edge, where bags develop occupies 1/12.5 of the entire cross-stream diameter. This implies that the Rayleigh–Taylor instability is the underlying reason for the local bag formation. Similar bag structures are also observed in the numerical simulation of diesel jet breakup at We = 1270 by Shinjo and Umemura (2010).

**Fig. 14:**
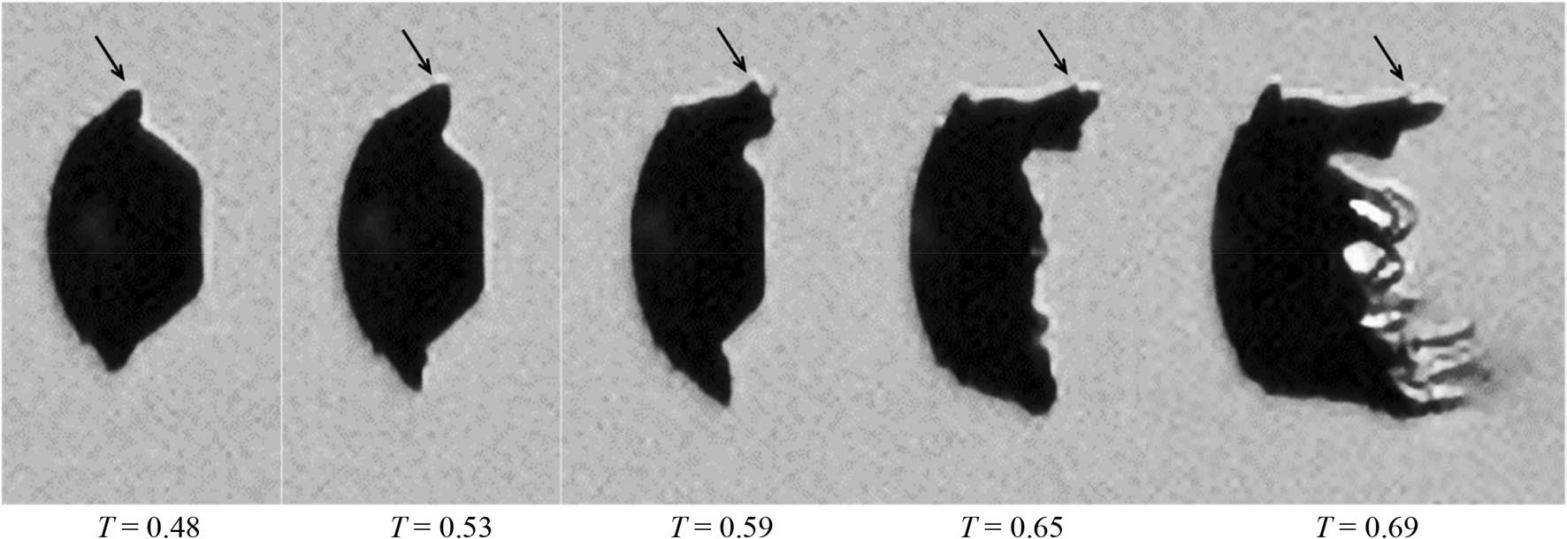
Folding of the liquid sheet (indicated by the arrows) and generation of ligaments connected by thin membranes for Case 5.1 at *M*_*∞*_ = 1.19

For Case 5.1 at *M*_∞_ = 1.19, the droplet breakup is characterized by the generation of long and thin streamwise ligaments in the wake, similarly to the observation by Liu and Reitz (1997). Figure 14 presents the deformation of the liquid sheet which induces the succeeding generation of ligaments. After emerging from the main body, the liquid sheet bends along the flow direction and folds to wrap the droplet rear before the breakup is initiated. The sheet becomes thinner as it stretches and thus is increasingly sensitive to instabilities. According to the study of sheet breakup in co-flow by Stapper and Samuelsen (1990), surface instability is mainly caused by the growth of the streamwise vortical waves when the flow velocity is high. As a result, streamwise ligaments connected by thin films are formed (*T* = 0.69 in Fig. 12). After the films burst into micro-drops, the ligaments are stretched under the viscous shear of the flow and break into larger fragments in the presence of Rayleigh-Plateau instabilities. The subsequent breakup of the droplet continues in the pattern that new ligaments form and fragment. It is occasionally observed that large liquid clumps (*T* = 1.3 in Fig. 12) detach from the intact body and disintegrate separately. It is noteworthy that recent studies (Jalaal and Mehravaran 2014; Meng and Colonius 2018; Biasiori-Poulanges and El-Rabii 2019; Dorschner et al. 2020) propose the mechanism of transverse azimuthal modulation as an alternative explanation for the generation of streamwise ligaments. It is stated that the growth of Kelvin–Helmholtz instabilities near the droplet periphery triggers Rayleigh–Taylor instabilities in the transverse plane which further lead to the formation of ligaments.

The breakup process of Case 5.2 is also presented in Fig. 12. The droplet exhibits few breakup features in common with the identical-Re Case 3.2, but resembles approximately the same characteristics as the identical-*M*_∞_ Case 5.1. A minor difference between Case 5.2 and Case 5.1 is that the former generates slightly thinner ligaments than the latter. This suggests that *M*_∞_ plays a dominant role in determining the breakup patterns, while Re only affects detailed structures.

**Fig. 15:**
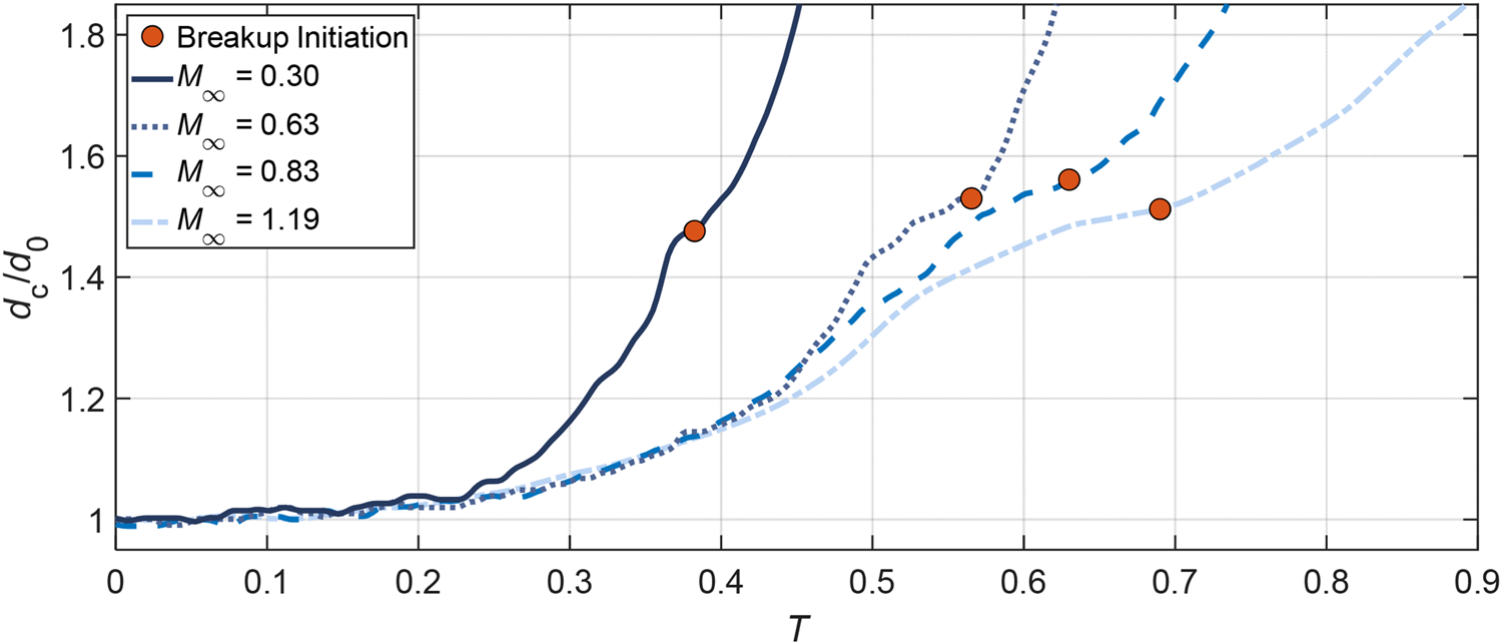
Variation of the cross-stream diameter for Case 1.4, 2.3, 3.2 and 5.1. The red dots represent the breakup initiation. The plotted data are averaged values from four repeated experiments

Figure 15 compares the change of the droplet cross-stream diameter *d*_*c*_ between subsonic and supersonic conditions. Before *T* = 0.25, the droplet deformation is characterized by the flattening of the main body. During this stage only marginal differences are identified among all cases. Afterwards the liquid sheet starts to develop, resulting in a rapid growth of *d*_*c*_. The growth rate is significantly higher for *M*_∞_ = 0.3 than the others, which is in accordance with the observation in Fig. 7. Although the differences between high-*M*_∞_ cases (Case 2.3, 3.2 and 5.1) are comparatively small, there exists a consistent tendency that the cross-stream diameter grows more slowly as *M*_∞_ increases. Once the breakup is initiated (marked by the red points in Fig. 15), *d*_*c*_ represents the cross-stream spread of the liquid fragments instead. With the breakup onset as a separating point, the overall *d*_*c*_ profile is divided into two power-law stages. The short-duration plateaus ahead of the breakup initiation correspond to the periods when the liquid sheet is bent along the flow direction rather than stretched out radially.

For all cases, the breakup begins when the normalized *d*_c_ reaches approximately 1.5. The subsonic Case 1.4 at *M*_∞_ = 0.3 experiences the earliest breakup, and the corresponding initiation time *T* = 0.39 matches the empirical correlation proposed by Pilch and Erdman (1987). The postponement of the breakup initiation as *M*_∞_ increases is a consequence of the change in the breakup pattern as shown earlier. The fragmentation of multiple bags and the disintegration of streamwise ligaments at high *M*_∞_ need significantly longer time than the direct sheet rupture at low *M*_∞_.

### Fragment sizes and spatial distributions

**Fig. 16:**
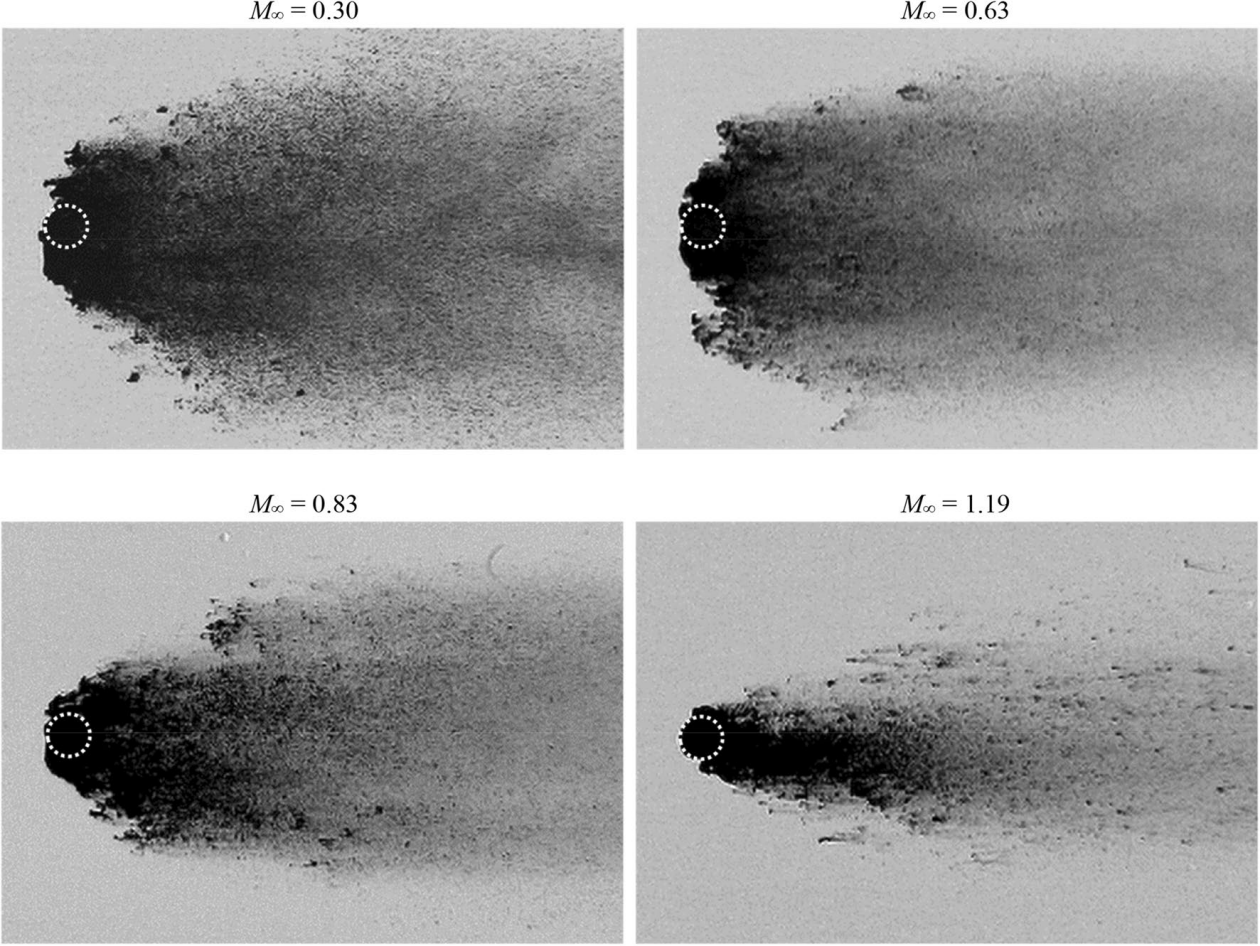
Spatial distributions of liquid fragments at *T* = 2.0 for Case 1.4, 2.3, 3.2 and 5.1. The white circles represent the initial droplet sizes

In industrial applications that involve atomization processes, the fragment sizes are of particular significance. For instance, small fragments are desired in fuel injections to achieve efficient evaporation and combustion. Figure 16 compares the droplet fragmentation at *T* = 2.0 for Case 1.4, 2.3, 3.2 and 5.1. At this stage, the coherent body is difficult to identify as the windward surface is severely eroded. Droplets break into uniformly fine mist at subsonic conditions, while the supersonic flow leads to large discrete particles that are scattered among tiny micro-drops. These large particles are mainly caused by the disintegration of ligament structures.

**Fig. 17:**
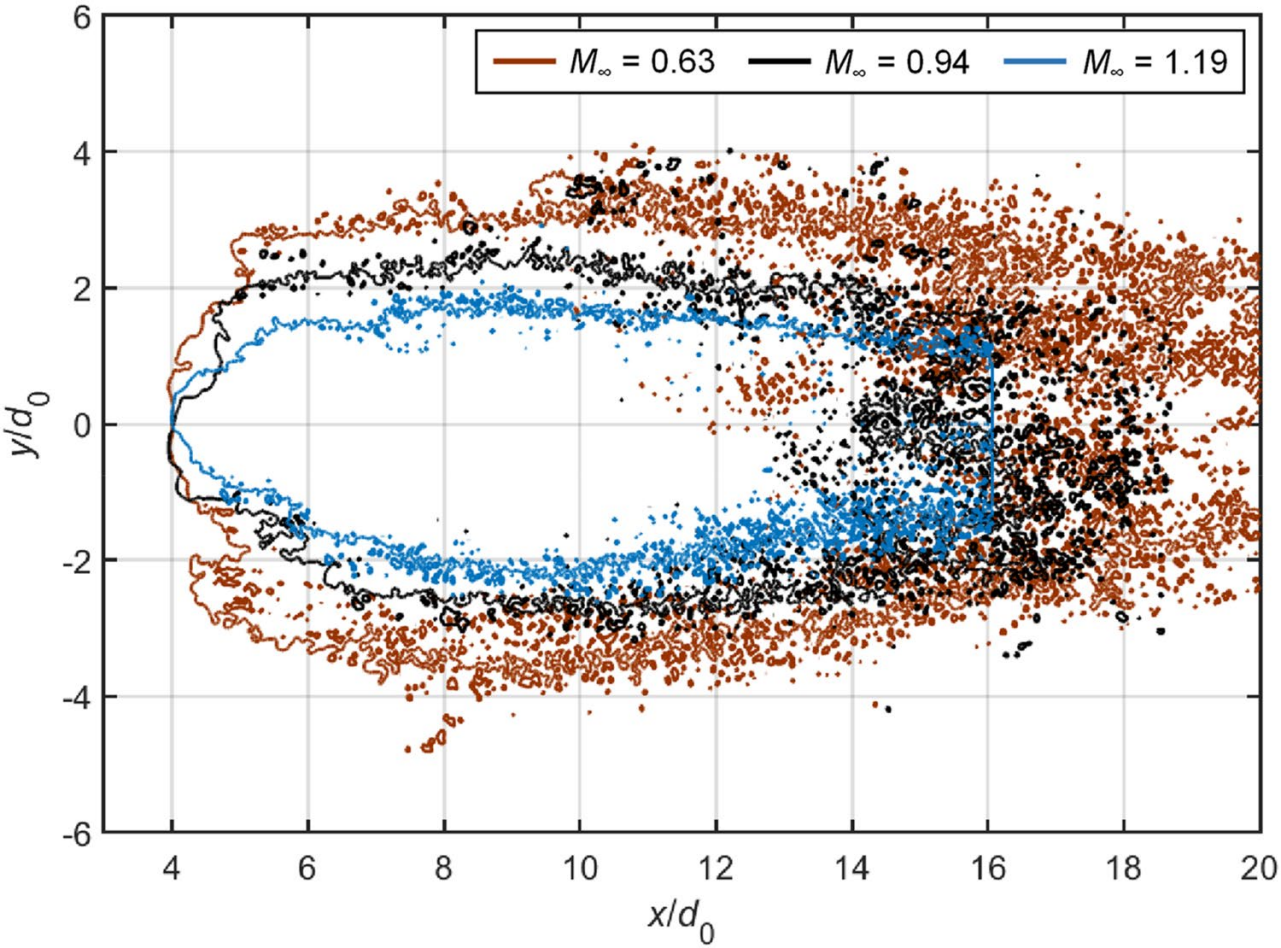
Comparison of fragment spreads at *T* = 2.0 between Case 2.3, 4.3 and 5.3. For all cases, Re is approximately 8.6e3

**Fig. 18:**
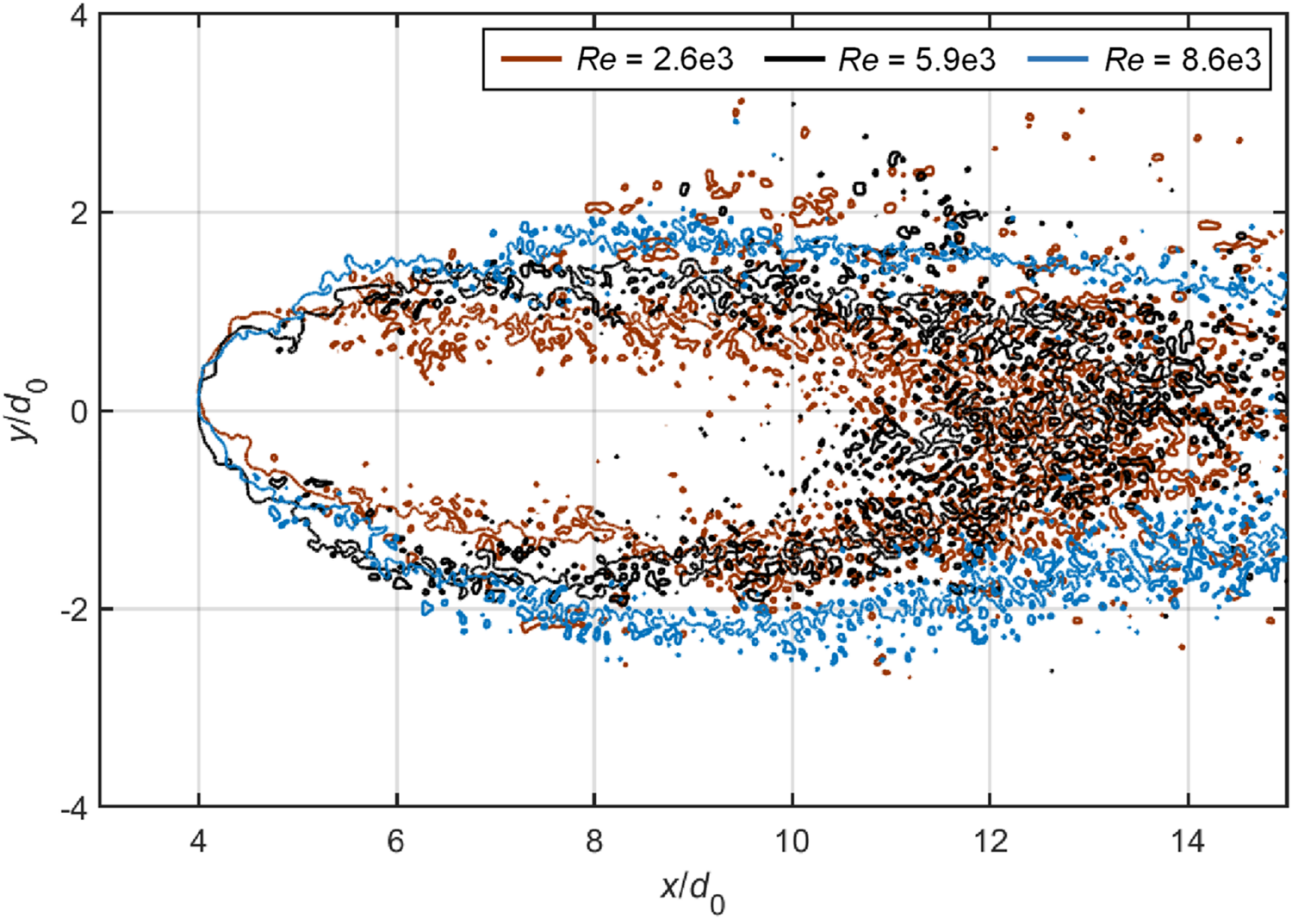
Comparison of fragment spreads at *T* = 2.0 between Case 5.1, 5.2 and 5.3. For all cases, *M*_*α*_ equals to 1.19

Apart from the size distribution, the spatial spread of the fragments is also of practical importance, since it determines the likelihood of micro-drops coalescing into larger particles. Figures 17, 18 present the outlines of dispersed fragments for various cases at *T* = 2.0. The outlines are extracted with MATLAB^®^ based on the modified Moore-Neighbor tracing algorithm (Gonzalez et al. 2004). The differences among Case 2.3, 4.3 and 5.3 (Fig. 17), which share the same Re, indicate that higher *M*_∞_ leads to a narrower cross-stream spread of the fragments. Such an effect could be related to the fact that the windward surface of the droplet is less flattened but more curved at higher *M*_∞_ (see Figs. 7, 12). Correspondingly, the fragments gain lower cross-stream momentum from the gas flow when detaching from the droplet periphery and are hence distributed less widely in the cross-stream direction. The comparison between Case 5.1, 5.2 and 5.3 (Fig. 18), which share an identical *M*_∞_, shows that lowering Re also tends to constrain the spatial distribution of the fragments, but much less effectively than increasing *M*_∞_.

### Effect of the Ohnesorge number

**Fig. 19:**
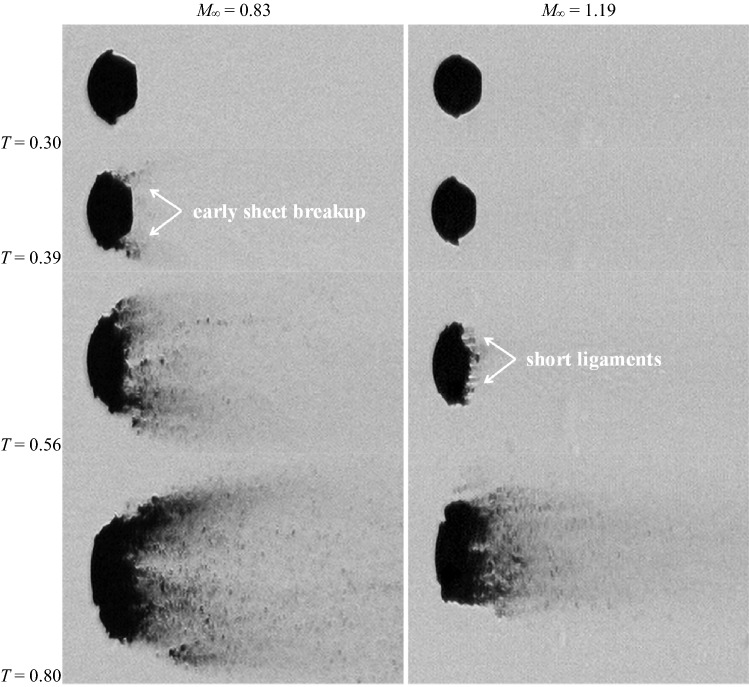
Breakup process of water droplets at *M*_*α*_ = 0.83 (Case 3.3) and 1.19 (Case 5.3)

Figure 19 displays the breakup process of water droplets at *M*_∞_ = 0.83 (Case 3.3) and *M*_∞_ = 1.19 (Case 5.3). Compared to ethylene glycol, water is much less viscous and yields a 20 times lower Ohnesorge number. The reduction in the liquid viscosity weakens the capability of sustaining large liquid sheets. Consequently the corresponding breakup initiations are much earlier than those of ethylene glycol droplets at the same *M*_∞_ (0.39 vs. 0.63 at *M*_∞_ = 0.83, 0.56 vs. 0.69 at *M*_∞_ = 1.19). Furthermore, in contrast to the long outstretched ligaments observed for ethylene glycol droplets in supersonic flows, the ligaments of water droplets extend over a very limited distance before disintegration (*T* = 0.56 at *M*_∞_ = 1.19). This is consistent with the observation by Stapper et al. (1992) that less viscous liquids sustain shorter ligaments.

## Conclusions

The present experimental work compares the stripping breakup of liquid droplets in subsonic and supersonic flows. Millimeter-sized droplets are impacted by a planar shock wave generated in a shock tube. The process of deformation and fragmentation is visualized in a shadow/schlieren system and recorded by an ultra-high-speed camera. Through controlling the shock strength and employing different liquids and gases, the Weber number is maintained around 1100, while the flow Mach number *M*_∞_ varies from 0.3 to 1.19 and the Reynolds number Re from 2600 to 24,000. The effects of changing flow conditions on the breakup process are summarized as follows.

- The droplet deformation prior to the breakup, including flattening of windward and leeward surfaces and growth of the liquid sheet at the equator, is weakened by increasing *M*_∞_. The sheet is initiated further downstream along the droplet surface at higher *M*_∞_.
- In subsonic flows, the stripping breakup starts with the fragmentation of the liquid sheet. As *M*_∞_ increases, distinct breakup features emerge at the breakup initiation, such as multiple bags formed along the periphery and ligament structures stretching in the wake. Correspondingly, the breakup initiation is significantly postponed.
- In subsonic flows, the breakup generates uniformly fine fragments spreading widely in the cross-stream direction. At higher *M*_∞_, the fragments are of less uniform sizes and constrained within a narrower region behind the main body.
- Although decreasing Re tends to have a complementary role to increasing *M*_∞_, the effect is marginal on main breakup behaviors.
- Lowering Oh reduces the size of liquid sheets and ligaments and results in earlier breakup initiation.

Although many features discussed in the present study focus on detailed breakup structures, they play crucial roles in determining the final fragmentation pattern. The dependency of fragment sizes and spatial distributions on the flow Mach number is especially important for industrial applications, where the atomization process is constantly optimized to generate widely-spread uniformly-sized fragments.
